# 1091. Validation of an Allometrically Scaled Body Weight Equation to Predict Vancomycin Clearance and Guide 24-Hour Vancomycin AUC Dosing in Obese Patients

**DOI:** 10.1093/ofid/ofab466.1285

**Published:** 2021-12-04

**Authors:** Brent Footer, Arthur Nguyen, Meagan Greckel, Colton Taylor, Alyssa Christensen, Gregory Tallman

**Affiliations:** 1 Providence Portland Medical Center, Portland, Oregon; 2 Providence Saint Vincent Medical Center, Portland, Oregon; 3 School of Pharmacy, Pacific University, Portland, Oregon

## Abstract

**Background:**

Accurately determining empiric vancomycin (VAN) doses in obese patients represents a clinical challenge. A recent population pharmacokinetic (PK) study provided an equation to estimate vancomycin clearance (CL) based on age, sex, serum creatinine (Scr), and allometrically scaled body weight. The purpose of this study was to validate this equation in a population of obese adults treated with vancomycin at eight community-based hospitals and use the CL estimate to guide empiric VAN dosing.

**Methods:**

The study period was November 1, 2020 and March 30, 2021. Patients were included if they were ≥ 18-year-old with a body mass index (BMI) ≥ 30 kg/m^2^, had an empiric dose targeting an AUC24 determined using the above referenced equation, and had a calculated AUC24. Only the first vancomycin course and AUC calculation for each patient were included. Patients with a creatinine clearance < 30ml/min and pregnant women were excluded. AUC24 and other PK parameters were calculated using two levels and noncompartmental analysis. Observed versus predicted CL and AUC24 were plotted to determine correlation.

**Results:**

Sixty patients were included, of which 60% were male and 33% had a confirmed methicillin-resistant *Staphylococcus aureus* infection. The mean age, BMI, and baseline Scr were 61.8 years, 37.8 kg/m^2^, and 0.99 mg/dL, respectively. Fifty-three (88%) patients received a loading dose, with a mean dose of 20.3mg/kg. The mean initial total daily maintenance dose was 2397.9mg. The mean predicted AUC24 was 476.4mg*h/L while the mean observed AUC24 was 556.3mg*h/L. For CL, the correlation between observed and predicted values was R^2^=0.38 (Figure 1). The correlation between predicted and observed AUC24 values was R^2^=0.08 (Figure 2). The percent of patients with observed AUC24 values of < 400mg*h/L, 400-600mg*h/L, and >600mg*h/L were 23%, 40%, and 37%, respectively. The relationship between calculated minimum concentrations (C_min_) and AUC24 is shown in Figure 3. 65% of patients with a therapeutic AUC achieved it with a C_min_ < 15mg/L while 4.5% of patients with a supratherapeutic AUC had a C_min_ < 15mg/L.

Figure 1. Observed versus Predicted Clearance (CL) of Vancomycin

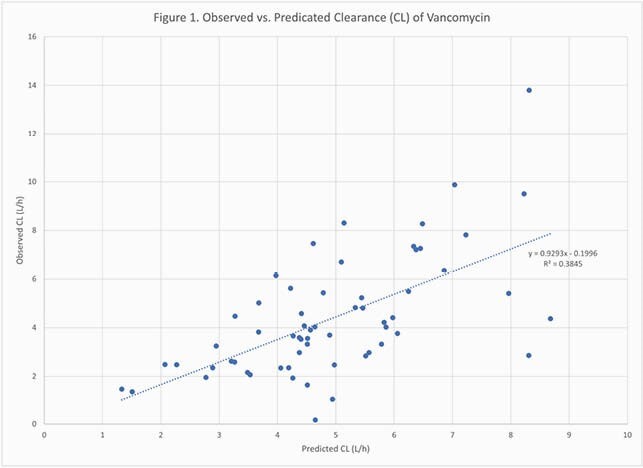

Figure 2. Observered versus Predicted 24 hour Vancomycin Area Under the Curve (AUC24)

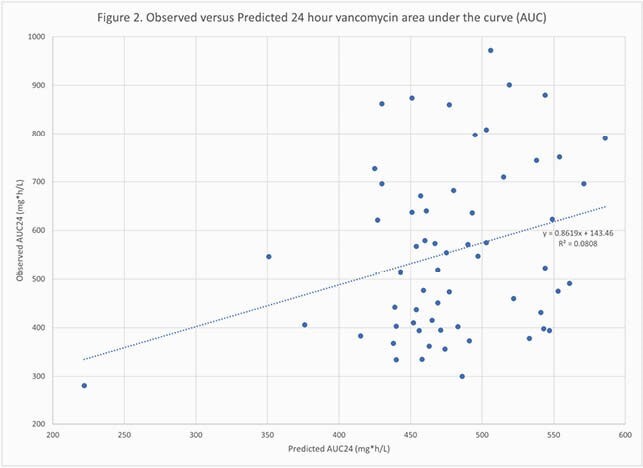

Figure 3. Calculated 24 hour Vancomycin Area Under the Curve (AUC24) versus Calculated minimal concentration (Cmin)

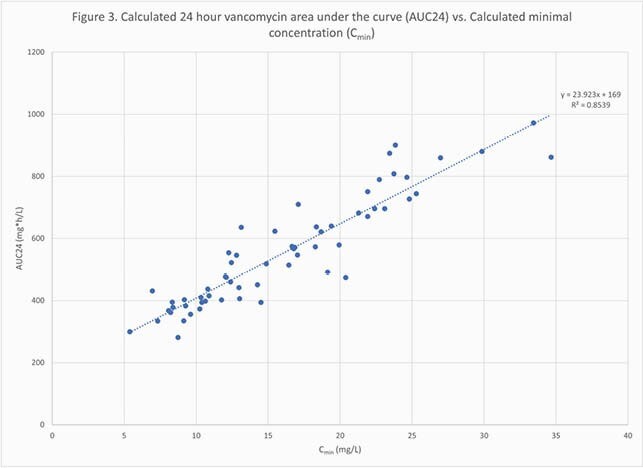

**Conclusion:**

The correlation between observed and predicted CL was 0.38. Using these CL estimates to guide empiric VAN dosing resulted in only 40% of patients achieving a therapeutic AUC24.

**Disclosures:**

**All Authors**: No reported disclosures

